# Testing the effect of early‐life reproductive effort on age‐related decline in a wild insect

**DOI:** 10.1111/evo.13679

**Published:** 2019-01-10

**Authors:** Rolando Rodríguez‐Muñoz, Jelle J. Boonekamp, Xing P. Liu, Ian Skicko, David N. Fisher, Paul Hopwood, Tom Tregenza

**Affiliations:** ^1^ Centre for Ecology and Conservation, School of Biosciences University of Exeter Penryn Campus TR10 9FE United Kingdom; ^2^ College of Forestry Jiangxi Agricultural University Nanchang 330045 Jiangxi China; ^3^ Department of Psychology, Neuroscience & Behaviour McMaster University 1280 Main St West Hamilton Ontario L8S 4L8 Canada

**Keywords:** Ageing, condition‐dependence, disposable soma, insects, senescence, trade‐off

## Abstract

The disposable soma theory of ageing predicts that when organisms invest in reproduction they do so by reducing their investment in body maintenance, inducing a trade‐off between reproduction and survival. Experiments on invertebrates in the lab provide support for the theory by demonstrating the predicted responses to manipulation of reproductive effort or lifespan. However, experimental studies in birds and evidence from observational (nonmanipulative) studies in nature do not consistently reveal trade‐offs. Most species studied previously in the wild are mammals and birds that reproduce over multiple discrete seasons. This contrasts with temperate invertebrates, which typically have annual generations and reproduce over a single season. We expand the taxonomic range of senescence study systems to include life histories typical of most temperate invertebrates. We monitored reproductive effort, ageing, and survival in a natural field cricket population over ten years to test the prediction that individuals investing more in early‐reproduction senesce faster and die younger. We found no evidence of a trade‐off between early‐life reproductive effort and survival, and only weak evidence for a trade‐off with phenotypic senescence. We discuss the possibility that organisms with multiple discrete breeding seasons may have greater opportunities to express trade‐offs between reproduction and senescence.

The disposable soma theory (Kirkwood 1977) provides a general explanation for the evolution of senescence. According to this theory, organisms cannot both maximize reproduction and maintain their bodies indefinitely. Reproduction therefore comes at the cost of a decline in physical condition and performance, ultimately leading to death (Kirkwood and Rose 1991). This hypothesized trade‐off between reproduction and survival makes clear predictions for experimental studies: imposition of increased reproductive effort should result in more rapid senescence and shorter lifespan, while experimentally relaxing reproductive effort is predicted to have the opposite effect. Likewise, manipulations of lifespan should have the inverse effect on reproductive effort. This prediction has been largely borne out by experimental studies on invertebrates in the lab (Zwaan et al. 1995; Hunt et al. 2006), with the exception of social insects (Monroy Kuhn and Korb 2016; Schrempf et al. 2017). It is also supported by the one experimental bird study where brood size was manipulated over multiple breeding seasons (Boonekamp et al. 2014) and a number of other studies on birds and mammals (Lemaître et al. 2015). However, a meta‐analysis of experimental studies on birds (Santos and Nakagawa 2012) found no overall effect of reproductive effort on adult survival.

The theory is difficult to test in unmanipulated natural systems. If mortality and reproductive effort are both condition‐dependent (Hoback and Wagner 1997; Furness and Reznick 2017; Ronget et al. 2017) selective mortality acting on poor condition individuals will tend to remove those individuals with low reproductive effort earlier in life, masking the existence of a trade‐off in correlational studies (Lemaître et al. 2015; Ronget et al. 2017). Moreover, species are likely to have evolved the capacity to optimize their investment in reproduction and somatic maintenance according to their phenotypic condition. Therefore, as in other life‐history traits where high‐condition individuals are able to express both traits involved in the trade‐off at a high level, we might expect a similar positive relationship between senescence and reproductive effort in the wild, rather than being able to observe the putative trade‐off (Van Noordwijk and De Jong 1986; Ronget et al. 2017).

It is therefore somewhat unexpected that many observational studies of wild populations do indeed appear to identify such trade‐offs (Lemaître et al. 2015). One possible explanation for the frequent detection of negative trade‐offs between reproductive effort and lifespan in nature may relate to the taxonomic bias in existing studies. Tests of the disposable soma theory in nature are strongly biased toward vertebrates, mainly birds and mammals (e.g., Santos and Nakagawa 2012; Lemaître et al. 2015; Bleu et al. 2016), with research on invertebrates being virtually absent. Nearly all vertebrates included in ageing studies have multiple breeding seasons (with rare exceptions; (Tozzini et al. 2013)). In such species, the relationship between investment in somatic maintenance and fitness and the equivalent relationship between investment in reproduction and fitness will have complex forms that relate to seasonal breeding opportunities. For instance, the fitness gained from surviving to just before the next breeding season will be much less than that from surviving a few months longer, until the end of that breeding season. The relationship between reproductive investment and lifespan may be more likely to reveal underlying trade‐offs in those species where individuals may reduce or completely avoid reproductive investment in one season to invest more in a subsequent season.

In species with multiple breeding seasons there is the potential for individuals to invest heavily in reproduction (at a cost to survival) in some seasons but not others according to individual condition and environmental factors. This opportunity for selection does not exist in species that live for only a single continuous breeding season. These single breeding season species have been described as extreme and unusual examples of condition‐independent mortality (Ronget et al. 2017), a characterization that seems inappropriate given how common univoltine life histories are in insects (Tauber et al. 1986). In temperate regions, insects often face a decline in the value of reproduction with time, due to the need to breed at a date that allows their offspring to survive the winter and reach adulthood at the optimal time the following year. Because any remaining energy not allocated to reproduction by the time the seasonal deadline is reached would be wasted, we might not expect to see the same patterns in these invertebrates as those observed in mammals and birds with multiple breeding seasons. The shape of the investment in reproductive effort as the season moves on will depend on the specific life history of the species: When successful reproduction is possible right up to the time of the seasonal deadline, individuals might increase their reproductive effort in the late part of the season to spend all their remaining energy just before dying, given they have no residual reproductive value (Williams 1966). However, if the optimum timing for successful reproduction peaks at some point in the season and then declines, reproductive effort should be maximal at the optimum date, and mainly constrained by condition.

The paucity of studies of senescence in wild insects is presumably mainly due to the difficulty of collecting longitudinal data from small, highly mobile animals. The field cricket *Gryllus campestris* provides a model system where individual tracking of adults is feasible due to their obligatory associations with burrows. Over the last 12 years, the *WildCrickets* project (www.wildcrickets.org) has monitored a wild population of these crickets in a meadow in North Spain. We have recorded physical and behavioral data for the whole lives of almost the entire adult population. This represents a uniquely comprehensive multigenerational study of a genetically characterized population (Bretman et al. 2011) providing detailed measurements of a host of naturally and sexually selected traits. These include male calling activity (below), encounters between individuals (Fisher et al. 2016b), matings, fights between males (Fisher et al. 2016a), predation events (Rodríguez‐Muñoz et al. 2011), and movement of individuals around their environment (Fisher et al. 2015). Here, we use these data to assess the existence of a trade‐off between investment in reproduction and somatic maintenance. Despite our reservations about the paradigm of anticipating a trade‐off in unmanipulated individuals, we follow the same approach that has dominated vertebrate studies to facilitate comparison between our study and the existing literature. We therefore test two complementary hypotheses in male crickets: individuals with a higher investment in reproductive traits in their early‐adult lives will (a) die earlier than those with a lower investment and (b) senesce faster in terms of the rate of decline in their expression of an energetically demanding trait.

## Methods

### STUDY SYSTEM

The WildCrickets meadow is located in North Spain, and is known to have been occupied by a natural population of the field cricket *G. campestris* for at least the last 45 years. The meadow is managed in a similar fashion every year. The grass is mowed once in mid‐March and once again in July–August; between August and March, the grass is kept short with additional mowing. Every year since 2006, weekly searches for burrows have been made from February until the end of the breeding season sometime in July, when the last adult cricket dies. Each burrow is flagged with a unique number that will identify it for the whole breeding season. By mid‐ to late‐April, usually before the adults start to emerge, we install up to 133 infrared day/night cameras (the number of cameras increased from an initial 64 in 2006), which continuously record the activity around each burrow entrance. The cameras are connected to several computers provided with motion activated digital video recording software (Diginet, dvr‐usa.com, replaced in 2011 with icatcher, icode systems.co.uk) so that video is only recorded when movement is detected around the burrow. Every individual is trapped a few days after emerging as an adult (see: crickettrapping.wordpress.com). Each one is weighed (± 0.01 g), photographed and marked with a PVC tag glued onto the pronotum, before being released back into the same burrow. The tag has a unique 1–2 character code, which allows each individual to be identified on the video. For every individual, we also collect a sample of cuticular hydrocarbons (by gently rubbing the pronotum with filter paper around 100 times). We also collect an approximately 10 μL drop of haemolymph (sampled by piercing the membrane at the hind leg joint), and a small piece of the tip of one of the hind legs (Rodríguez‐Muñoz et al. 2010). These samples are later used to provide individual pheromone and DNA profiles.

Adult crickets regularly move around the meadow occupying different burrows. At the beginning of the season the number of occupied burrows is often greater than the number of cameras. We carry out direct observations to cover non‐videoed burrows by directly observing the occupants of every burrow that lacks a camera every 1–4 days. We record the ID of any adult present or whether a nymph is in residence. This allows us to accurately record adult emergence dates even in burrows that are not video monitored at that particular time; last instar nymphs and recently emerged adults rarely move between burrows, so the presence of an adult where there was a nymph the day before indicates an emergence. Videos are stored on a RAID server and watched using iCatcher software developed specifically for our project. After the end of the season, we watch the videos and record all significant events (particularly: adult emergence, encounters between individuals, singing activity, matings, fights and their outcome, oviposition, predator attacks, movement of individuals around the meadow). A weather station installed in the center of the meadow logs weather variables at ten minutes intervals, including measurements from three additional temperature sensors located on the surface of the meadow and four in simulated burrows, at locations scattered around the meadow. The project has been running between 2006 and 2017, although years 2014 and 2017 are still being processed.

### REPRODUCTIVE INVESTMENT (EFFORT) VARIABLES

Emergence date: In our study populations adult emergence is highly synchronized in both sexes, with 95% of the male population emerging as adults every year within a period of two weeks (annual emergence period over 10 years for 95% of the male population = 15 ± 3.53 days, mean ± SD). Adult emergence marks the point when an insect switches from growth to reproduction. Earlier emergence allows males to begin mating earlier and may be advantageous for their offspring who have longer to develop before the end of the warm summer months.

Calling activity: Male crickets sing from outside their burrows to attract potential mates. We quantified calling activity for each male by recording whether he was singing or not over the first 10 minutes of every hour. For those ten minutes, at one minute intervals we noted whether the male was singing or not. If any of those ten samples was positive, then the cricket was recorded as singing in that hour. If singing was not observed for any of the ten samples, he was recorded as not singing. For each studied male, this measure provided up to 24 binary samples per day throughout its life (depending on how much his burrow was monitored). Only samples where the male was alone at the burrow and at least five days old were included. To reduce noise due to small sample size, only days with five or more samples for any given male and males with at least 24 samples in total were included in the analysis.

Searching activity: Male crickets also find potential mates by visiting different burrows around the meadow. To score the intensity of this searching activity, we used the duration of visits that each individual made to burrows; shorter stays are associated with more frequent moves between burrows. The duration of burrow visits is extremely right skewed, ranging from less than 1 min to over 10 days with a median of 77 min. To analyze these skewed data, we transformed searching activity into a binary variable by classifying burrow stays as either short (≤ 77 min, representing intensive searching) or long (> 77 min, representing lower searching activity). We coded short stays as 1 and long stays as 0 to place more actively searching males in the high scoring group for consistency with the other measures. Because stay duration can be influenced by the presence of another cricket at the same burrow, we only included visits where the focal male was on his own for the duration of the whole visit.

Dominance in fights: Males frequently encounter other males at burrows. When this happens, either one of them leaves the area immediately, or they engage in a fight (typically lasting for only a few seconds) for the occupation of the burrow. Burrow occupancy allows the male the opportunity to mate with any female using the burrow, so a male's success in these fights is a key measure of his dominance. Although each fight only produces a single independent piece of data (there is always a winner and a loser) there are many more fights than there are males, so for every fight, both participants received a score of either 1 of 0 according to whether they won or lost, and that score was recorded against the age of the male.

Mating promptness: Typically, at some point after a pair meets, the male will begin courting the female. The time taken from their initial encounter to when a pair mate has a very right skewed distribution. Most matings occur within the first few minutes, but latencies can be over 40 hours. Because of this extreme skew, we transformed latency into a binary variable, classifying intervals of ≤ 50 min as (1) and those of > 50 min as long (0) (hence the higher score represents faster mating). The 50 minute threshold was chosen because it is the minimum time that we have measured between consecutive matings by a male, and a similar refractory period has been measured in laboratory assays on the closely related *G. bimaculatus* (Sakai et al. 1995). This presumably reflects the time required to produce a new fully formed spermatophore, so males failing to mate for longer than 50 minutes have had sufficient time to do so, even if they arrived at the burrow without a spermatophore they had already produced.

### STATISTICAL ANALYSES

#### Detection of senescence

We analyzed the effect of age on four reproductive behaviors expressed throughout the breeding season using a longitudinal modeling approach that allowed us to partition out the effects of age within individuals from the effects of the average age over which individuals were sampled. The method has been previously described in detail (Van de Pol and Wright 2009). In short, we divide age into the average age over which an individual was sampled and the difference between this average age and their age at a particular sampling point (delta age). Hence, delta age (*Δage*) reflects within‐individual changes with age, removing the effect of selective disappearance. We interpret negative associations between *Δage* and trait expression as evidence for reproductive senescence.

Before dividing age into its two components, we selected the relevant variables by running mixed models of different complexity and selecting the one with the best fit to the data. For each of the response variables, the full model (the most complex model) included fixed effects of age, quadratic age and temperature (except for the probability of winning a fight, as temperature is the same for both contenders), and year and cricket identity as random intercept factors. We also included the difference in age and size (pronotum width) between the two males as additional fixed effects in our analysis of fights, and female age as a fixed effect in our analysis of mating promptness (to account for potential changes in female willingness to mate with age). Reproductive traits often show a quadratic relationship with age, increasing early in life, and decreasing after a certain age of peak performance. We therefore tested for quadratic relationships between traits and age by including also a quadratic term of age. When the best model included this quadratic relationship, we specifically considered the postpeak decline of *Δage* to reflect senescence (see below). We ran those models fitted by maximum Likelihood using the *lme4* R package, and considered a model as the best supported when its AIC value was at least seven units smaller than that of the following simpler model (Burnham et al. 2011). When the best model for a trait included the quadratic term, we estimated the age of peak performance using the recently developed threshold model approach (Douhard et al. 2017). An accurate estimate of the peak age is important because it has a strong effect on the estimation of the postpeak age trajectory (Rodríguez‐Muñoz et al. unpubl. ms.). We use the same threshold methods as in our recent study (Rodríguez‐Muñoz et al. unpubl. ms.). In short, threshold models divide age into two periods, pre‐ and postpeak age periods, such that their sum equals the lifespan. A range of peak ages (thresholds) is then tested and the best‐supported peak age and its confidence interval can be evaluated by comparing their AIC values. As described above for model selection, we took a conservative approach and considered peaks within seven AICs to indicate a lack of difference between them (Burnham et al. 2011). We only identified quadratic relationships (i.e., those where the quadratic term of age was included in the model with the best fit) for calling activity. We ran threshold models for calling activity for each year independently and detected a clear peak in calling activity every year apart from 2008 (which had the lowest population size) (see also (Rodríguez‐Muñoz et al. unpubl. ms.)). Peak ages varied among years (from 12 to 19 days postadult emergence). To combine data across years we took the mean peak age across the 8 years where a clear peak was available resulting in an overall peak age of 15 days.

#### Detection of life‐history trade‐offs

For the detection of life‐history trade‐offs, we investigated the relationships between early‐adult‐life reproductive investment and late‐adult‐life performance measured through survival and trait expression. We made the distinction between early‐ and late‐adult life stages based on the peak age of calling activity. This was the only trait for which we could identify a clear peak in performance, and it is also known to be energy demanding (Hoback and Wagner 1997), making it a useful trait in the context of ageing. Hence, “early‐life” refers to prepeak adult ages and “late‐life” to postpeak adult ages in relation to peak age in male calling activity. As measures of late‐life adult performance we used post‐peak survival probability and postpeak decline in calling activity. We then tested the effects of early‐life reproductive investment by classifying each individual male in one of two groups having a high/low reproductive investment score, depending on whether he was above or below the population median for each of the five reproductive traits described above (emergence date, calling activity, searching activity, dominance in fights, and mating promptness). To this end, we ran a Cox proportional‐hazards regression for each trait, to test the effect of early‐adult life reproductive investment (high/low) on postpeak survival probability using the R *survival* package (Therneau 2015). Likewise, we tested the effect of early‐adult life reproductive investment (high/low) on the decline in calling activity postpeak. Specifically, we tested the interaction between the factor denoting high/low prepeak investment and postpeak *Δage* to identify whether postpeak age trajectories of calling activity depended on prepeak reproductive investment. Because we used decline in calling activity as a measure of senescence, we did not include the same trait as a measure of early‐life reproductive effort in this senescence analysis, as higher activity early in life would provide more scope for subsequent declines. We considered using random slope models for these analyses by including the random interaction term of individual identity x postpeak *Δage*. However, random slopes are only accurate when the age of peak calling activity can be estimated on the individual level. Such individual estimates can be computed by running threshold models with individual random slopes, but these were either too demanding computationally, or failed to converge properly. Consequently, we used random intercept models to determine the overall effect of reproductive effort on postpeak calling activity.

## Results

### SENESCENCE

**Figure 1:**
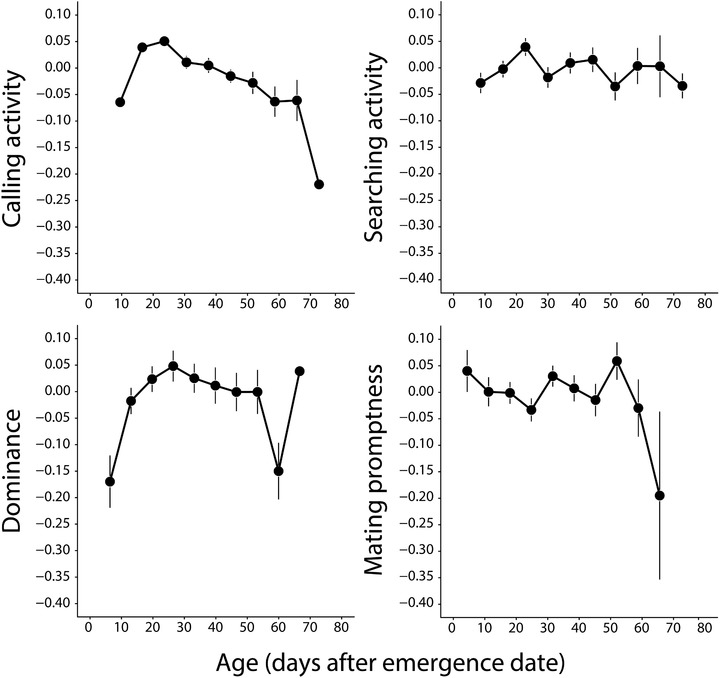
Age trajectories of four male reproductive traits: calling activity, dominance, mating promptness, and searching activity. To capture the relationship between these traits and within‐individual age we used the residuals from a model including environmental temperature and average age (apart from dominance where environmental temperature is irrelevant as both males in a fight are at the same temperature). Data points and error bars reflect the mean of residual trait values combined in 7‐day bins and their respective standard errors. Note that our statistical analyses were done with the raw data, that is with temperature as covariate and without binning of age. Points at young ages where no error bars are visible are due to errors smaller than the diameter of the point. The oldest ages in the calling activity and dominance figures are a single individual and hence no error can be calculated.

We found evidence for senescence in two of the four analyzed traits, calling activity, and dominance in fights, as shown by the significance of the within individuals age effect (Fig. 1, Table 1). Calling activity was the only trait showing a quadratic relationship with age (Table S1). When we analyzed the postpeak dataset, we found a decline in calling activity indicated by the significant negative effect of age within individuals (Fig. 1, Table 1). Male dominance in fights also declined significantly with age within individuals (Table 1). Relative size and age of the focal male as compared to the opponent had a strong positive effect on the probability of winning a fight (Table 1). We found no effect of age within‐individuals on searching activity and mating promptness (Fig. 1, Table 1).

**Table 1:** Relationship between age and four reproductive traits in wild *Gryllus campestris* males

Fixed effects		Calling activity	Searching activity	Dominance in fights	Mating promptness
	**Est**	−5.480	−5.510	0.010	−4.961
**Intercept**	**SD**	0.108	0.195	0.074	0.312
	***P***	***< 0.001***	***< 0.001***	0.892	***< 0.001***
	**Est**	0.283	0.305	—	0.357
**Temperature**	**SD**	0.004	0.009	—	0.018
	***P***	***< 0.001***	***< 0.001***	—	***< 0.001***
	**Est**	—	—	0.046	—
**Age difference**	**SD**	—	—	0.011	—
	***P***	—	—	***< 0.001***	—
	**Est**	—	—	6.335	—
**Size difference**	**SD**	—	—	0.408	—
	***P***	—	—	***< 0.001***	—
	**Est**	−0.113	−0.039	−0.164	−0.089
**Age within‐individuals**	**SD**	0.017	0.041	0.069	0.075
	***P***	***< 0.001***	0.340	***0.018***	0.235
	**Est**	0.062	−0.223	0.162	−0.008
**Age among‐individuals**	**SD**	0.072	0.067	0.097	0.070
	***P***	0.385	***0.001***	0.095	0.906
**Number of samples**		53,293	7,993	2,456	3,205
Random effects					
	**Var**	0.438	0.351	0.624	0.123
**Individual**	**SD**	0.662	0.592	0.790	0.351
	***N***	327	422	373	372
	**Var**	0.056	0.067	—	—
**Year**	**SD**	0.237	0.259	—	—
	**N**	9	9	**—**	—

We decomposed age into ***Δage*** (within‐individuals effects) and mean age (among‐individuals effects) (***Age*** = **μ*Age*** + ***ΔAge***, see Van de Pol and Wright 2009). *A*mbient temperature (*Temperature*) and the difference in age (*Age difference*) and size (*Size difference*) between fighting contenders (only for dominance in fights) are included as fixed effects. **M**ale identity (*ID*) and year (*Year*) are included as random effects. For calling activity, the analysis uses the ages after the peak in calling activity (15d). The table shows the results of a mixed model per trait using the *lme4* R package (Bates et al. 2014) with a binomial error distribution. **Est**, coefficient estimation: **SD**, standard deviation; **Var**, variance. Coefficients with significant *P* values are highlighted in bold italics.

### LIFE‐HISTORY TRADE‐OFFS

**Figure 2:**
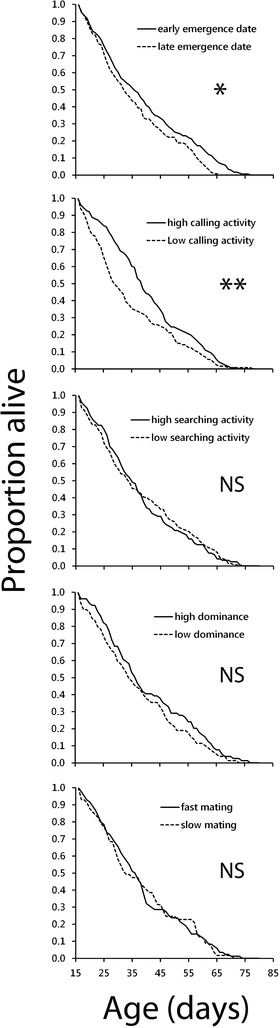
Cumulative survival through late adult life comparing among two groups classified according to early investment in five reproductive traits in wild *Gryllus campestris* males. We classified individuals into each of the two groups according to their adult emergence date (before or after the median emergence date) or their early‐adult‐life trait‐investment in calling activity, intensity of active female searching, dominance in fights against other males, and how promptly they mate when finding a female. Early‐adult life was defined as the period from 0–15 d, during which calling activity increased postmaturity before subsequently decreasing in late life (from 15 d old). Lines represent those above (continuous) or below (broken) the population median for investment in each trait (for emergence, continuous = early emergence). Early‐adult investment was associated with a significant effect on subsequent survival for emergence date and calling activity, and had no effect for any of the other traits (Cox's Proportional–Hazards regression).

Males that emerged earlier than the median emergence date in the season (which we interpret as an earlier switch from growth to reproductive investment) survived for longer than those that emerged later (Cox proportional‐hazards regression, *Wald's z* = 2.55, *P* = 0.011, *N* = 296, see Fig. 2). Similarly, males that spent more time calling than the median before reaching the age of the peak in calling activity, also had higher survival (*Wald's z* = 2.66, *P* = 0.008, *N* = 253, see Fig. 2). We found no effect of early investment in searching activity, dominance in fights, and mating promptness on survival after the age of 15 days (Fig. 2).

**Figure 3:**
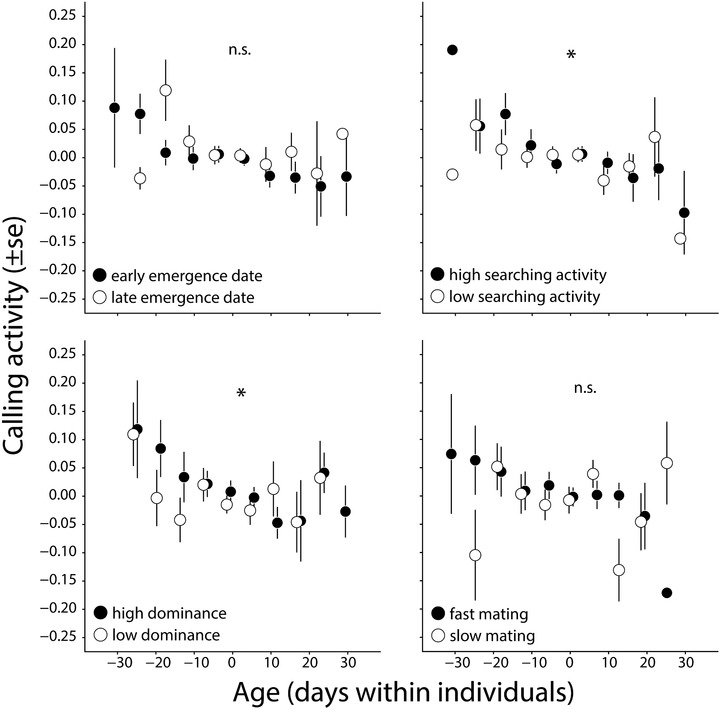
Effect of early‐life (prepeak) investment in four reproductive traits on the postpeak age trajectory of calling activity. For each trait, we classified males into high or low effort groups, according to whether their investment was above or below the median. Data points and error bars reflect the mean of residual calling activity over temperature per 7‐day age bins, as also explained in Fig. 1. Asterisks denote a significant interaction between prepeak reproductive effort and postpeak age‐related decline in calling activity. Points where no error bars are visible are due to errors smaller than the diameter of the point.

The model including the interaction between reproductive investment in early life and the subsequent within individual decline in calling activity had the lowest AIC for all the measures of reproductive investment, except mating promptness, although none of these AICs was more than 7 units smaller than the AIC of the simplest model (which did not include reproductive investment, see Table 2). However, the model including the interaction between age within individuals and reproductive investment was very close to having a better fit than the model without it (ΔAIC = 6) for two of the traits, searching activity and dominance in fights. The output of both models showed that the interaction between reproductive investment in early‐adult life and the subsequent decline in calling activity is significant (Table 3, Fig. 3). Males that were more active in searching for females or more dominant in early life (before the age of 15 d), showed a steeper decline in calling activity in late life (after the age of 15 d) than less dominant or active searching males, suggesting the existence of a trade‐off between investment in reproduction and body maintenance.

**Table 2:** Model comparison testing the effect of early‐life investment (before the age of peak calling activity) in four reproductive traits on the rate of decline in calling activity in late life (after the age of peak calling activity) in wild *Gryllus campestris* males

		ΔAIC			
Model	Df	*Emergence date*	*Searching activity*	*Dominance in fights*	*Mating promptness*
*Sings ∼ Temp* + dAge*RI + *MeAge* + (*1* | *ID*) *+* (*1* | *Year*)	8	−3	−6	−6	4
*Sings ∼ Temp* + *dAge* + *MeAge* + *RI* + (*1* | *ID*) *+* (*1* | *Year*)	7	1	2	1	2
*Sings ∼ Temp* + *dAge* + *MeAge* + (*1* | *ID*) *+* (*1* | *Year*)	6	0	0	0	0

The full model includes ambient temperature (*Temp*), age within (*dAge*) and among (*MeAge*) individuals as fixed effects, and male identity (*ID*) and year (*Year*) as random effects. The table shows the difference in AIC for each model as compared to the simplest model with the smallest AIC (differences in AIC < 7 are considered as nonsignificant; Burnham et al. 2011). All models have been analyzed using the *lme4* R package (Bates et al. 2014) with a binomial error distribution.

**Table 3:** Effect of early‐life investment (before the peak age in calling activity) in two reproductive traits on the relationship between age and late life‐calling activity (after the peak age in calling activity) in wild *Gryllus campestris* males

Fixed effects		Searching activity	Dominance in fights
	**Est**	−5.405	−5.368
**Intercept**	**SD**	0.119	0.147
	***P***	***< 0.001***	***< 0.001***
	**Est**	0.279	0.282
**Temperature**	**SD**	0.004	0.006
	***P***	***< 0.001***	***< 0.001***
	**Est**	−0.192	−0.163
**Age within‐individuals**	**SD**	0.025	0.030
	***P***	***< 0.001***	***< 0.001***
	**Est**	−0.009	−0.099
**Low reproductive investment**	**SD**	0.098	0.123
	***P***	0.926	0.421
	**Est**	0.104	0.114
**Age among‐individuals**	**SD**	0.077	0.108
	***P***	0.179	0.290
	**Est**	0.108	0.128
**Age within‐ind x Low rep. inv**.	**SD**	0.034	0.043
	***P***	***0.001***	***0.003***
**Number of samples**		38,976	21,883
Random effects			
	**Var**	0.413	0.373
**Individual**	**SD**	0.657	0.610
	***N***	233	123
	**Var**	0.039	0.036
**Year**	**SD**	0.197	0.189
	***N***	9	9

The model includes *Δage* (within‐individuals effects), mean age (among‐individuals effects) (*Age* = *μAge* + *ΔAge*, see Van de Pol and Wright 2009), ambient temperature and the interaction between the score in reproductive investment and age within individuals as fixed effects, and male identity (ID) and year (Year) as random effects. The table shows the results of a mixed model per trait using the *lme4* R package (Bates et al. 2014) with a binomial error distribution. Est., coefficient estimation: SD, standard deviation; Var. variance. Coefficients with significant *P* values are highlighted in bold italics.

## Discussion

Our analyses reveal extensive variation in the reproductive effort of males and in how this effort varies with age in wild field crickets. There was convincing evidence of senescence in singing activity and dominance but not in how much individuals moved around the meadow (searching activity) or in how rapidly a male mated with a female after they met. We have previous reported increasing probability of mortality with age in this species (Rodríguez‐Muñoz et al. unpubl. ms.), and similar patterns of actuarial senescence have been described across a range of wild insects including another field cricket species *Teleogryllus commodus* (Zajitschek et al. 2009a), the antler fly (*Protopiophila litigate*) (Bonduriansky and Brassil 2005), and the Neriid fly*Telostylinus angusticollis* (Kawasaki et al. 2008), several species of lepidopterans (Carroll and Sherratt 2017) and the damselfly *Coenagrion puella* (Sherratt et al. 2010). In contrast, evidence of phenotypic senescence is less consistent, and our study, despite having an unusually large and detailed dataset, only identified phenotypic senescence in two of the traits we studied–calling activity and dominance.

Phenotypic senescence has been observed in *T. commodus* males, where calling activity of long‐living individuals increased during an early period of their lives, reached a peak and then declined (Zajitschek et al. 2009b). This study did not find evidence of a trade‐off between reproduction and survival, with both showing a positive relationship. In *P. litigate*, Bonduriansky and Brassil (2005) found evidence of both a decline in mating rate with age, and a trade‐off between reproduction and survival: males that mated more often in early‐life senesced faster and had a lower survival. However, in the damselfly *C. puella*, neither of these effects was found; mating activity did not decline with age, and individuals mating more often did not die earlier (Hassall et al. 2015). In our study, mate searching ability and promptness to mating did not show any consistent age‐related pattern of expression. Our findings are based on a very large number of observations including more than ten years of data, suggesting that we are unlikely to have missed a strong pattern of declining performance with age. However, whether the lack of senescence in two out of four traits was because these traits genuinely do not show any biologically significant changes in expression over the lifespan or whether it is just that the tremendous amount of environmental noise impinging on these animals in nature obscures these effects is something we can only speculate about.

Our investigation of potential trade‐offs between early‐adult life investment and subsequent declines in survival and performance produced similarly equivocal results. When we measured reproductive investment as how early males switched from the growth (nymph) phase to the reproductive (adult) phase, or as how much time they spent singing in the first part of their adult lives, there were positive associations between these measures of early‐reproductive investment and lifespan. This is the direct opposite of the prediction of a trade‐off. In contrast, when comparing early‐life reproductive investment measured as searching activity or dominance there was some evidence of a trade‐off with the subsequent decline in the rate of singing. The best‐fitting model included the interaction between early‐life reproduction and within‐individual age, and it explained a significant proportion of the variance (Table 3) suggesting that our study should be regarded as consistent with the existence of a trade‐off. We note that we cannot be very confident in this observation because the difference in AICc values between the models of senescence with and without the interaction with early‐adult life reproductive investment was < 7 (Table 2; although the appropriate threshold AIC difference is still debated; Burnham et al. 2011). Inspection of Figure 3 illustrates the uncertainty around these interactions: for the comparison between high searching and low searching males there is a clear pattern of senescence in the high searching group and no change with age in the low searching group. However, in the comparison of high and low dominance males, the differences between senescence in these groups are harder to identify from the figure. We also analyzed these data by coding dominance as a continuous trait, rather than creating high/low dominance groups that gave very similar results (in this case the difference in AIC values was larger, indicating a greater improvement of fit) but this approach is very hard to visualize.

The modest evidence for a trade‐off in some traits and no evidence for a trade off in others contrasts with the pattern described from most field studies on unmanipulated female birds and mammals (Lemaître et al. 2015). It is however, consistent with the findings of many studies on male birds and mammals (Bleu et al. 2016) and with most experimental bird studies (Santos and Nakagawa 2012). As touched upon in our introduction, a potential general explanation for the difference between our study population and many studies of other wild populations could be that the expression of these trade‐offs is related to the iteroparous life histories of the long‐lived vertebrates that have been studied previously. Multiannual breeding species have the potential to defer investment in reproduction in one season in anticipation of investing more in a subsequent season. They also have large eggs or young and males frequently need to compete with other males to monopolize females. This may mean that individuals have to choose between investing very heavily in a given breeding season, or else investing very little. This dichotomy may mean that in a particular breeding season there is the opportunity to express a trade‐off between reproduction and survival, which differs from the scenario in single‐breeding season species such as our crickets.

For most annual insects, investment in reproduction is continuous throughout the breeding season and no individual survives to a second‐breeding period. As a result, if there are diminishing returns from investment in reproductive traits and somatic maintenance traits, then individuals in good condition and optimizing investment will invest more in both survival and reproduction and individuals in poor condition will invest less in both these outcomes. This is the classic car‐house paradox (Van Noordwijk and De Jong 1986), whereby individuals in good condition invest in several costly traits in parallel, masking the existence of negative correlations between them. The specific pattern of investment in reproduction with age in these annual species will depend on the relationship between fitness and the timing of reproduction. Presumably for most species there will be some optimal date for reproduction to occur to match local environmental conditions and to provide offspring with sufficient time to develop to adulthood in the next season. This is likely to be the case for species like *G. campestris*, where the optimum time for reproduction is probably close to the start of the breeding season. However, *G. campestris* females presumably face physiological constraints that prevent them from developing and laying all their eggs simultaneously. There may also be some potential for competition between newly hatched nymphs that might expand the optimal window for egg laying. Nevertheless, we expect females to concentrate as much of their reproductive effort as possible around the optimum laying date, and to decline due to somatic deterioration as the season moves forward after that optimum time. This is clearly an area ripe for theoretical development, perhaps utilizing a dynamic state variable approach along the lines of that applied to oviposition behavior in egg‐limited females (Mangel and Heimpel 1998).

The weak evidence for a realized trade‐off in our system also differs from the findings of laboratory experiments on another field cricket, *Teleogryllus commodus*. In this species, males fed a high protein diet sang more often and died earlier than those fed low protein food (Hunt et al. 2004). Similarly, males from lines of *T. commodus* selected for long lifespan called less than males selected in the opposite direction (Hunt et al. 2006). The difference between our study and these lab studies may reflect differences between these superficially similar species. It is noteworthy that *T. commodus* appears to have the capacity to enter winter diapause or not, depending on the population origin (Hogan 1965), whereas our study species has obligatory diapause. Another possible explanation for the observation of a trade‐off in *T. commodus* that was not observed in *G. campestris* may be related to the relative potential for a trade‐off to be detected. It might be that the very strong manipulations applied in these experimental studies reveal trade‐offs that are not apparent when only natural variation in individual condition is present.

In summary using our ten generations of data we were able to identify senescence in some energetically costly reproductive behaviors in males (calling activity and dominance), but not in others (mate searching and mating promptness). We failed to detect any evidence for trade‐offs between investment in early reproduction and lifespan. Instead we found that greater early‐life reproductive investment tended to be associated with longer lifespan, suggesting both are dependent on condition. In contrast to this positive association, there was some evidence for trade‐offs between early investment in some reproductive traits (particularly dominance and searching activity) and the rate of decline in calling activity later in life. Our results provide evidence for senescence in multiple traits in a wild, univoltine invertebrate. They also suggest that there may be realized trade‐offs between reproductive investment and somatic maintenance, although these appear to be much weaker than those identified in wild vertebrates.

## CONFLICT OF INTEREST

The authors do not have any conflict of interest.

Associate Editor: S. Sumner

Handling Editor: M. Servedio

## Supporting information


**Table S1**. Model selection for the relationship between age and four reproductive investment bivariate traits in wild *Gryllus campestris* males.

## Data Availability

The data used for this study will be archived in the UK Natural Research Environmental Council data repository. The Environmental Information Data Centre http://eidc.ceh.ac.uk/ before publication.
