# Supplementary material for: Testing the effect of early‐life reproductive effort on age‐related decline in a wild insect
Source: Evolution. 2019 Jan 10;73(2):317–28. doi: 10.1111/evo.13679 (PMC6590129; doi:10.1111/evo.13679)
Supplement: Supplementary file 1 — Table S1. Model selection for the relationship between age and four reproductive investment bivariate traits in wild Gryllus campestris males. [file EVO-73-317-s001.pdf]

Supporting information

Table S1. Model selection for the relationship between age and four reproductive investment bivariate traits in wild *Gryllus campestris* males. All full models include age (*Age*) and a quadratic term of (Age^2) as fixed effects, and individual (*ID*) and year (*Year*) as random effects. At least one of the following fixed factors is included depending on the trait: *Temp*, ambient temperature; *AgeDiff*, difference in age of the focal male minus his opponent; *SizeDiff*, difference in body mass of the focal male minus his opponent; *FemAge*, age of the mating female. The table shows the difference in AIC for each model as compared to the model with the minimum value scored as 0. Differences in AIC <7 are considered as non-significant (Burnham et al. 2011). All models have been analysed using the *lme4* R package (Bates et al. 2014) with a binomial error distribution. The best model is highlighted in bold.

Model comparison for each reproductive investment variable	Df	ΔAIC
Calling activity (Sings = calling or not when sampled)		
***Sings ~ Temp + Age + Age*² + (*1* I *ID*) + (*1* I *Year*)**	6	0
Sings ~ Temp + Age + (*1* I *ID*) + (*1* I *Year*)	5	1,692
Sings ~ Temp + Age + (*1* I *ID*)	4	1,699
Sings ~ Age + (*1* I *ID*)	3	11,994
Searching activity (*Short* = having a short (≤77 min) or long (>77 min) stay at burrow)		
*Short ~ Temp + Age + Age*² + (*1* I *ID*) + (*1* I *Year*)	6	2
***Sings ~ Temp + Age* + (*1* I *ID*) + (*1* I *Year*)**	5	0
Sings ~ Temp + Age + (*1* I *ID*)	4	18
Sings ~ Age + (*1* I *ID*)	3	1,379
Dominance in fights (*Won* = winning a fight)		
*Won ~ AgeDiff + SizeDiff + Age + Age*² + (*1* I *ID*) + (*1* I *Year*)	7	-4
Won ~ AgeDiff + SizeDiff + Age + (*1* I *ID*) + (*1* I *Year*)	6	2
***Won ~ AgeDiff + SizeDiff + Age* + (*1* I *ID*)**	5	0
Won ~ SizeDiff + Age + (*1* I *ID*)	4	16
Won ~ Age + (*1* I *ID*)	3	340
Mating promptness (*Short* = mating promptly (≤50 min) or not (>50min)		
*Short ~ Temp + FemAge + Age + Age*² + (*1* I *ID*) + (*1* I *Year*)	7	1
Short ~ Temp + FemAge + Age + (*1* I *ID*) + (*1* I *Year*)	6	0
Short ~ Temp + Age + (*1* I *ID*) + (*1* I *Year*)	5	-1
***Short ~ Temp + Age* + (*1* I *ID*)**	4	0
Short ~ Age + (*1* I *ID*)	3	513

References

Bates, D., M. Mächler, B. Bolker, and S. Walker. 2014. Fitting linear mixed-effects models using lme4. arXiv preprint arXiv:1406.5823.

Burnham, K. P., D. R. Anderson, and K. P. Huyvaert. 2011. AIC model selection and multimodel inference in behavioral ecology: some background, observations, and comparisons. Behavioral Ecology and Sociobiology 65:23-35.
